# Atiprimod blocks STAT3 phosphorylation and induces apoptosis in multiple myeloma cells

**DOI:** 10.1038/sj.bjc.6602637

**Published:** 2005-06-21

**Authors:** M Amit-Vazina, S Shishodia, D Harris, Q Van, M Wang, D Weber, R Alexanian, M Talpaz, B B Aggarwal, Z Estrov

**Affiliations:** 1 Department of Bioimmunotherapy, The University of Texas M.D. Anderson Cancer Center, Houston, TX, USA; 2 Department Lymphoma/Myeloma, The University of Texas M.D. Anderson Cancer Center, Houston, TX, USA

**Keywords:** multiple myeloma, signal transduction, clonogenic assay, nuclear factor κB, apoptosis

## Abstract

Multiple myeloma (MM) accounts for 1 % of all cancer deaths. Although treated aggressively, almost all myelomas eventually recur and become resistant to treatment. Atiprimod (2-(3-Diethylaminopropyl)-8,8-dipropyl-2-azaspiro[4,5] decane dimaleate) has exerted anti-inflammatory activities and inhibited oeteoclast-induced bone resorption in animal models and been well tolerated in patients with rheumatoid arthritis in phase I clinical trials. Therefore, we investigated its activity in MM cells and its mechanism of action. We found that Atiprimod inhibited proliferation of the myeloma cell lines U266-B1, OCI-MY5, MM-1, and MM-1R in a time- and dose-dependent manner. Atiprimod blocked U266-B1 myeloma cells in the G_0_/G_1_ phase, preventing cell cycle progression. Furthermore, Atiprimod inhibited signal transducer and activator of transcription (STAT) 3 activation, blocking the signalling pathway of interleukin-6, which contributes to myeloma cell proliferation and survival, and downregulated the antiapoptotic proteins Bcl-2, Bcl-X_L_, and Mcl-1. Incubation of U266-B1 myeloma cells with Atiprimod induced apoptosis through the activation of caspase 3 and subsequent cleavage of the DNA repair enzyme poly(adenosine diphosphate-ribose) polymerase. Finally, Atiprimod suppressed myeloma colony-forming cell proliferation in fresh marrow cells from five patients with newly diagnosed MM in a dose-dependent fashion. These data suggest that Atiprimod has a role in future therapies for MM.

Multiple myeloma (MM) is a clonal B-cell neoplasm characterised by an accumulation of neoplastic plasma cells. Multiple myeloma accounts for 1% of all cancer deaths and affects approximately 14 500 Americans each year (Jemal *et al*, 2003). For many years, intermittent courses of melphalan and prednisone constituted the standard therapy for MM. Several other drugs, including alkylating agents, vinca alkaloids, and, in recent years, thalidomide and its derivatives, and the proteasome inhibitor bortezomib, have been studied with various degrees of success (Dimopoulos *et al*, 2003; Anderson, 2004). However, almost all myelomas eventually recur and become resistant to treatment (Dimopoulos *et al*, 2003). Thus, the search for more effective agents continues.

Several studies have demonstrated that of all B-cell-stimulating factors, interleukin (IL)-6 plays the most important role in MM by regulating the growth and survival of the neoplastic cells (Kawano *et al*, 1988; Klein *et al*, 1995; Cote *et al*, 2002). Interleukin-6 has been implicated in the pathogenesis of MM (Klein *et al*, 1995). This cytokine, which is produced by both myeloma and bone marrow (BM) stroma cells, stimulates MM cell proliferation in an autocrine and paracrine fashion (Klein *et al*, 1995; Chauhan *et al*, 1996), augments osteoclast activity (Szczepek *et al*, 2001), and provides the neoplastic cells with a survival advantage (Lichtenstein *et al*, 1995). Indeed, serum IL-6 levels in patients with MM have been shown to be correlated with disease stage, activity, and survival (Klein *et al*, 1990). Thus, the inhibition of IL-6 activity may be an adequate strategy for suppressing MM cells.

Atiprimod (SK&F 106615; Callisto Pharmaceuticals, New York, NY, USA) is an azaspirane, a cationic amphiphilic compound, with anti-inflammatory activity. Several azaspiranes have been found to be beneficial in animal models of adjuvant induced arthritis (Bradbeer *et al*, 1996), autoimmune encephalitis (Badger *et al*, 1989), lupus erythematosus (Albrightson-Winslow *et al*, 1990), autoimmune diabetes mellitus (Rabinovitch *et al*, 1993), and solid organ transplantation graft rejection (Badger *et al*, 1991; Fan *et al*, 1993). Although azaspiranes have been studied extensively, their mechanism of action is not completely understood. Several mechanisms, including the upregulation of nonspecific (Badger *et al*, 1990a, 1990b) and splenic (Badger *et al*, 1990a, 1990b; Schmidbauer *et al*, 1993) suppressor cell activity and downregulation of various cytokine receptors (e.g. IL-2 receptor, transferrin receptor), adhesion molecules (e.g., ICAM-1), and cytokines (e.g. IL-2, interferon-*γ*, IL-1, tumour necrosis factor-*α*, and IL-6) have been reported (Schmidbauer *et al*, 1993). These mechanisms and the ability of azaspiranes to normalise bone mineral density, prevent structural damage to joints and periarticular bone, and downregulate serum IL-6 levels (Bradbeer *et al*, 1996) without inducing myelotoxicity (King *et al*, 1991) suggest that these compounds may be effective against autoimmune disorders and diseases causing bone destruction. Therefore, Atiprimod was administered to patients with rheumatoid arthritis in phase I clinical trials and found to be well tolerated.

Owing to Atiprimod's broad array or activities, we speculated that Atiprimod would inhibit MM cells by suppressing the activity of IL-6. We found that Atiprimod blocked the activation of the signal transducer and activator of transcription (STAT) 3, inhibited myeloma cell proliferation, and induced cell cycle arrest and apoptosis in MM cells.

## MATERIALS AND METHODS

### Patients

Bone marrow (BM) aspirates were obtained from five patients with newly diagnosed MM (Table 1). All studies were performed with the patients’ informed consent and were approved by the Institutional Review Board at The University of Texas M.D. Anderson Cancer Center, Houston, TX, USA.

### Cell lines

The MM cell lines MM-1, U266-B1, and RPMI-8226 were obtained from the American Type Culture Collection (ATCC; Rockville, MD, USA), MM-1R cells (a dexamethazone-resistant variant of the MM-1 line) were provided by Steven Rosen (Northwestern University Medical School, Chicago, IL, USA), and the cell line OCI-MY5 was provided by Hans Messner (Ontario Cancer Institute, Toronto, ON, Canada). MM-1, MM-1R, and U266-B1 cell lines were maintained in RPMI 1640 (Sigma Chemical Co., St Louis, MO, USA) supplemented with 10% fetal calf serum (FCS; Hyclone, Logan, UT, USA). OCI-MY5 cells were maintained in Iscove's modified Dulbecco's medium (IMDM) supplemented with 10% FCS. The KM102 marrow stroma cell line was established from normal BM (Harigaya and Handa, 1985) and maintained in RPMI 1640 supplemented with 10% FCS.

### Drug preparation

Atiprimod (Callisto Pharmaceuticals, New York, NY, USA) was dissolved in phosphate-buffered saline (PBS; GIBCO BRL, Grand Island, NY, USA) at a final concentration of 8 mM. The stock solution was further diluted in tissue culture medium.

### Enzyme-linked immunosorbent assay (ELISA)

Enzyme-linked immunosorbent assays were performed with IL-6 and IL-6 receptor (IL-6R) ELISA kits (Biosource International, Camarillo, CA, USA). In one experiment, U266-B1 cells were harvested at the logarithmic phase of their growth, washed twice in RPMI 1640, and incubated in RPMI 1640 supplemented with 10% FCS at 37°C with or without 2 *μ*M Atiprimod. Supernatant samples were harvested at 1, 6, 8, and 16 h after incubation. In another experiment, myeloma cell lines were incubated for 72 h with or without either normal marrow stroma or KM102 stroma cells in the presence or absence of Atiprimod. Supernatant samples were harvested and both IL-6 and soluble IL-6R levels were analysed in accordance with the manufacturer's instructions. Briefly, supernatant samples were added to the wells in duplicate and incubated for 2 h at 37°C. The test wells were then washed three times in PBS, incubated with rabbit IL-6 antiserum for 2 h, washed as previously described, and incubated for 30 min with goat anti-rabbit IgG conjugated to horseradish peroxidase. The test wells were vigorously washed, a substrate, provided by the manufacturer, was added, and the colour intensity was read within 15 min at a wavelength of 490 nm with a microplate reader.

### Electrophoretic mobility shift assay for analysis of NF-*κ*B activation

NF-*κ*B activation was analysed by electrophoretic mobility gel shift assay (EMSA) as described previously (Chaturvedi *et al*, 2000). In brief, 8-*μ*g nuclear extracts prepared from treated or untreated U266-B1 cells were incubated with a ^32^P-end-labelled 45-mer double-stranded NF-*κ*B oligonucleotide from the human immunodeficiency virus-1 long terminal repeat (5′-TTGTTACAAGGGACTTTCCGCT GGGGACTTTCCAG GGAGGCGTGG- 3′) for 15 min at 37°C, and the DNA–protein complex was resolved on a 6.6 % native polyacrylamide gel. The radioactive bands from the dried gels were visualised and quantitated by a PhosphorImager (Molecular Dynamics, Sunnyvale, CA, USA) with the ImageQuant software program.

### Western immunoblotting

Cell lysates were assayed for their protein concentration using the BCA protein assay reagent (Pierce Chemical, Rockford, IL, USA). Each set of paired lysate samples was then adjusted to have the same protein concentration. Sodium dodecyl sulphate–polyacrylamide gel electrophoresis (SDS–PAGE) (Laemmli, 1970) was conducted at constant wattage (10 W) in running buffer cooled to 4°C. Stacking gels contained 4% (wt vol^−1^) acrylamide, and separating gels contained 12% (wt/vol) acrylamide. Approximately 50 *μ*g of sample protein was loaded into each lane. Proteins separated with SDS–PAGE were transferred to nitrocellulose membranes; the transfers were performed overnight at 30 V in a cooled (4°C) reservoir containing 25 mM Tris (tris (hydroxymethyl) aminomethane), 192 mM glycine, and 20% methanol (pH 8.3) (Towbin *et al*, 1979) transfer buffer. The nitrocellulose membranes were then removed from the blot apparatus and placed in a solution of Ponceau S stain (0.5% Ponceau S and 1% glacial acetic acid in water) to verify equal loading of protein in the control and treated samples (Gershoni and Palade, 1983).

After membranes were stained for 5 min, they were rinsed for 2 min and examined. Equal loading of protein was verified, and the membranes were then rinsed for an additional 10 min and immunoscreened. The membranes were blocked with BLOTTO (5% dried milk dissolved in 50 ml of PBS) for at least 1 h at room temperature. They were then washed three times in PBS plus 0.5% Tween 20. Next, the membranes were incubated for 1 to 12 h with the appropriate antibodies. After incubation, the membranes were rinsed three times in PBS containing 0.5% Tween 20 for 15 min each. The bound antibody was detected with the ECL Western blotting detection system (Amersham, Arlington Heights, IL, USA). The membranes were incubated with an anti-rabbit and anti-mouse horseradish peroxidase-labelled antibody at a concentration of 1 : 200 and 1 : 1750, respectively, in PBS plus 0.5% Tween 20 at room temperature for 1 h. After this incubation, the membranes were washed in PBS containing 0.5% Tween 20, and bound antibody was detected using the ECL protocol. Chemiluminescence of the membranes was detected with X-OMAT AR5 X-ray film (Kodak, Rochester, NY, USA) in stainless steel exposure cassettes (Sigma).

The following antibodies were used: monoclonal mouse antihuman CPP32 (Trasduction Laboratories, Lexington, KY, USA) for detection of procaspase 3, rabbit anti-human cleaved caspase 3 (New England Bio Labs, Beverly, MA, USA), mouse anti-human poly(ADP-ribose) polymerase (PARP; Pharmingen, San Diego, CA, USA), mouse anti-human Bcl-2 (Transduction Laboratories, Lesington, KY, USA), rabbit anti-human Bcl-X_L_ (Transduction Laboratories), mouse anti-human Mcl-1 (Pharmingen), and mouse anti-human STAT3 and pSTAT3 antibodies (Upstate Cell Signaling Solutions, Charlottesville, VA, USA). Normal mouse immunoglobulin G (IgG) and rabbit IgG (Sigma) were used as controls. To confirm the detection of these proteins, we used lysates of Jurkat cells (ATCC) for the detection of procaspase 3, PARP, STAT3, pSTAT3, and Bcl-2; Hela cells (ATCC) for the detection of cleaved caspase 3; and human endothelial cells for the detection of Bcl-X_L_.

### Electrophoretic mobility shift assay for STAT3-DNA binding

The STAT3-DNA binding was analysed by EMSA using a ^32^P-labelled high-affinity sis-inducible element (hSIE) probe as previously described (Yu *et al*, 1995). Briefly, 2 × 10^6^ U266-B1 cells were incubated with 8 *μ*M Atiprimod for 0.5, 1, 2, 4, and 8 h. Nuclear extracts were prepared and labelled with hSIE probe (5′-CTTCATTTCCCGTAAATCCCTAAAGCT-3′ and 5′-AGCTTTAGGGATTTACGGGAAATGA-3′), and STAT3-DNA binding was analysed using EMSA as described above.

### MTT assay

The 3,(4,5-dimethylthiazol-2-yl)-5-(3-carboxymethoxyphenyl)-2-(4-sulfophenyl)-2*H*-tetrazolium (MTT) assay was performed using an MTT-based cell proliferation/cytotoxicity assay system (Promega, Madison, WI, USA). Briefly, U266-B1, MM-1, MM-1R, OCI-MY5, and RPMI-8266 cells were harvested at the logarithmic phase of their growth and fresh low-density MM marrow cells (containing >40% myeloma cells) were isolated by gradient centrifugation. They were then washed twice in RPMI 1640 containing 10% FCS and counted in a hemocytometer, and their viability was determined using 0.1% trypan blue staining. Equal numbers of viable cells (5 × 10^4^ cells per well) were incubated in a total volume of 100 *μ*l of RPMI 1640 medium supplemented with 10% FCS alone or with Atiprimod at increasing concentrations; the incubations were continued for 72 h in 96-well flat-bottomed plates (Linbro; Flow Laboratories, McLean, VA, USA) at 37°C in a humidified 5% CO_2_ atmosphere. In another experiment, the cells were incubated for 24, 48, and 72 h. After incubation, 20 *μ*l of CellTiter96 One Solution Reagent (Promega) was added to each well. The plates were then incubated for an additional 60 min at 37°C in a humidified 5% CO_2_ atmosphere. Immediately after incubation, absorbance was read using a 96-well plate reader at a wavelength of 490 nm. Each data point was determined six times before analysis.

### Cell cycle analysis

Cell cycle analysis was performed according to standard protocols. Briefly, 5 × 10^6^ cells were pelleted after incubation with Atiprimod. The cell pellets were washed and resuspended in 2 ml of 1% paraformaldehyde in PBS. Cells were incubated for 15 min at 4°C and then washed again in PBS, resuspended in 2 ml of absolute ethanol, and stored at −20°C until staining. Next, the stored cells were washed twice in PBS, resuspended in 0.5 ml of propidium iodide (PI) staining buffer (50 *μ*g/ml PI and 10 mg/ml RNase in PBS), and then incubated for 1 h at room temperature in total darkness. Flow cytometric analysis was performed using a FACSCalibur flow cytometer and the CellQuest software program (Becton Dickinson Immunocytometry Systems, San Jose, CA, USA). Data analysis was performed using CellQuest and the Modfit LT V2.0 software program (Verity Software House, Topsham, ME, USA).

### Apoptosis assays

To quantify the percentage of cells undergoing apoptosis, we used annexin V-CY5 (Pharmingen, San Diego, CA, USA) as previously described (Vermes *et al*, 1995). Briefly, Atiprimod-treated U266 cells were washed twice with cold PBS and then resuspended in binding buffer (10 nM HEPES (*N*-2-hydroxyethylpiperazine-*N*-2-ethanesulphonic acid), 140 nM NaCl, and 5 nM CaCl_2_, pH 7.4) at a concentration of 1 × 10^6^ cells *μ*l^−1^. After incubation, 100 *μ*l of the solution was transferred to a 5-ml culture tube to which 5 *μ*l of annexin V-CY5 and 10 *μ*l of PI were added. The tube was gently vortexed and incubated for 15 min at room temperature in total darkness. At the end of the incubation, 400 *μ*l of binding buffer was added to the tube, and the cells were analysed immediately by flow cytometry. Flow cytometric analysis was performed with a FACSCalibur flow cytometer using CellQuest. Data analysis was performed with CellQuest and Modfit LT V2.0.

To establish that Atiprimod induces apoptosis in MM cells, we used the TdT-mediated dUTP nick-end labelling (TUNEL) apoptosis detection system (Promega) as previously described (Estrov *et al*, 1999). Briefly, U266 cells were incubated with 6 *μ*M Atiprimod for 2 h in the presence or absence of a 50 *μ*M concentration of the caspase inhibitor Ac-DEVD-CHO (Thornberry *et al*, 1994) (CalBiochem, La Jolla, CA, USA). Formaldehyde-treated cytospun cells were made permeable with 0.2% Triton X-100 in PBS. After being washed, the slides were treated with equilibration buffer (supplied with the TUNEL kit) and then incubated with TdT buffer (prepared according to the manufacturer's instructions) for 60 min. The staining reaction was terminated by treating the slides with 2 × standard sodium citrate for 15 min. After another washing, the slides were treated with antifade solution and then mounted on slides with glass coverslips and rubber cement. The slides were analysed using a fluorescence microscope.

### Clonogenic assay

A modification of a previously described clonogenic assay was used to grow MM colony-forming cells (Millar *et al*, 1988; Rhodes *et al*, 1990). Briefly, fresh, low-density BM cells from patients with MM were fractionated using immunomagnetic beads, and 1 × 10^5^ CD3-, CD33-, and CD14-negative cells were cultured in 0.8% methylcellulose (Fluka Chemical, Ronkonkoma, NY, USA), containing 10% FCS and RPMI 1640 medium in 1% (vol vol^−1^) phytohemagglutinin (PHA) T-cell-conditioned medium, and irradiated feeder cells obtained from the low-density fraction of normal donor peripheral blood cells exposed to 70 Gy of *γ*-irradiation, as previously described (Estrov *et al*, 1994). The culture mixture was placed in 35-mm Petri dishes (Nunc, Naperville, IL, USA) in duplicate and maintained at 37°C with 5% CO_2_ in air in a humidified atmosphere. Colonies, which were defined as clusters of more than 40 cells, were counted after 7 days using an inverted microscope.

To verify the neoplastic nature of the colony-forming cells, single colonies were microaspirated; the rearranged immunoglobulin H (IgH) gene in their DNA was amplified by polymerase chain reaction (PCR), and the product was directly sequenced and compared to that of the diagnostic BM DNA as previously described (Estrov *et al*, 1994). To avoid contamination by residual effete myeloma cells, only isolated colonies were microaspirated. The rearranged IgH gene in the DNA from the diagnostic myeloma BM cells and from individual colonies was amplified using a modification of a previously described method (Deane and Norton, 1990). Briefly, cells were placed in 1 ml of QuickExtract DNA Extraction solution 1.0 (Epicentre, Madison, WI, USA) and lysed in accordance with the manufacturer's protocol. The DNA was harvested, and its concentration adjusted with nuclease-free water. Genomic DNA (1–1.5 *μ*g) was added to the reaction mixture, which contained 10 mM dNTP (Promega), 25 mM MgCl_2_ (Proemga), 2.5 U of *Taq* polymerase, *Taq* polymerase buffer (Promega), 50 pM upstream and downstream primers, and nuclease-free water in a total volume of 50 *μ*l. A separate tube was prepared for each of the five primers chosen from the first framework region of the coding strand of representative germline V_H_ family members amplified together with a J_H_ consensus primer of the IgH gene. The tubes were denaturated at 94°C for 5 min and then subjected to 35 cycles of denaturation at 90°C for 1 min, annealing at 66°C for 2 min, with an extension at 72°C for 2 min, and a final extension at 72°C for 5 min in the Techgene Thermal Cycler (Techne Inc., Princeton, NJ, USA). A sample of the amplified DNA was electrophresed in a 2% E-Gel (Invitrogen Corp., Carlsbad, CA, USA) and visualised by UV illumination. The bands of interest were excised, and the DNA was purified using the Wizard SV Gel and PCR Clean-Up System (Promega). Next, the DNA from the bands was sequenced using a DNA sequencer (Applied Biosystems, Foster City, CA, USA). The primer for the coding strand was the appropriate V_H_ family primer, and the noncoding strand was the J_H_ consensus primer.

## RESULTS

### Atiprimod suppresses IL-6 production by MM cells

Atiprimod (Atiprimod) was found to downregulate IL-6 production (Bradbeer *et al*, 1996). Therefore, we asked whether Atiprimod would also inhibit the production of IL-6 in MM cells. We incubated U266-B1 cells with or without 8 *μ*M Atiprimod and measured IL-6 supernatant levels using an ELISA. Consistent with previous reports (Catlett-Falcone *et al*, 1999) we found that U266-B1 cells produce IL-6, and that the levels of IL-6 in U266-B1 cell supernatant increased over time. Interleukin-6 supernatant levels increased from 28 pg ml^−1^ at 1 h after incubation to 71, 60, and 222 pg ml^−1^ at 6, 8, and 16 h, respectively. Atiprimod attenuated IL-6 supernatant levels to 23, 42, 53, and 130 pg ml^−1^ at 1, 6, 8, and 16 hours, respectively. Similar results were obtained when U266-B1 cells were incubated in the presence of normal stroma cells or the stroma cell line KM102 (Table 2). Thus, Atiprimod suppresses production of IL-6 by U266-B1 cells.

### Atiprimod blocks the activation of NF-*κ*B

**Figure 1:**
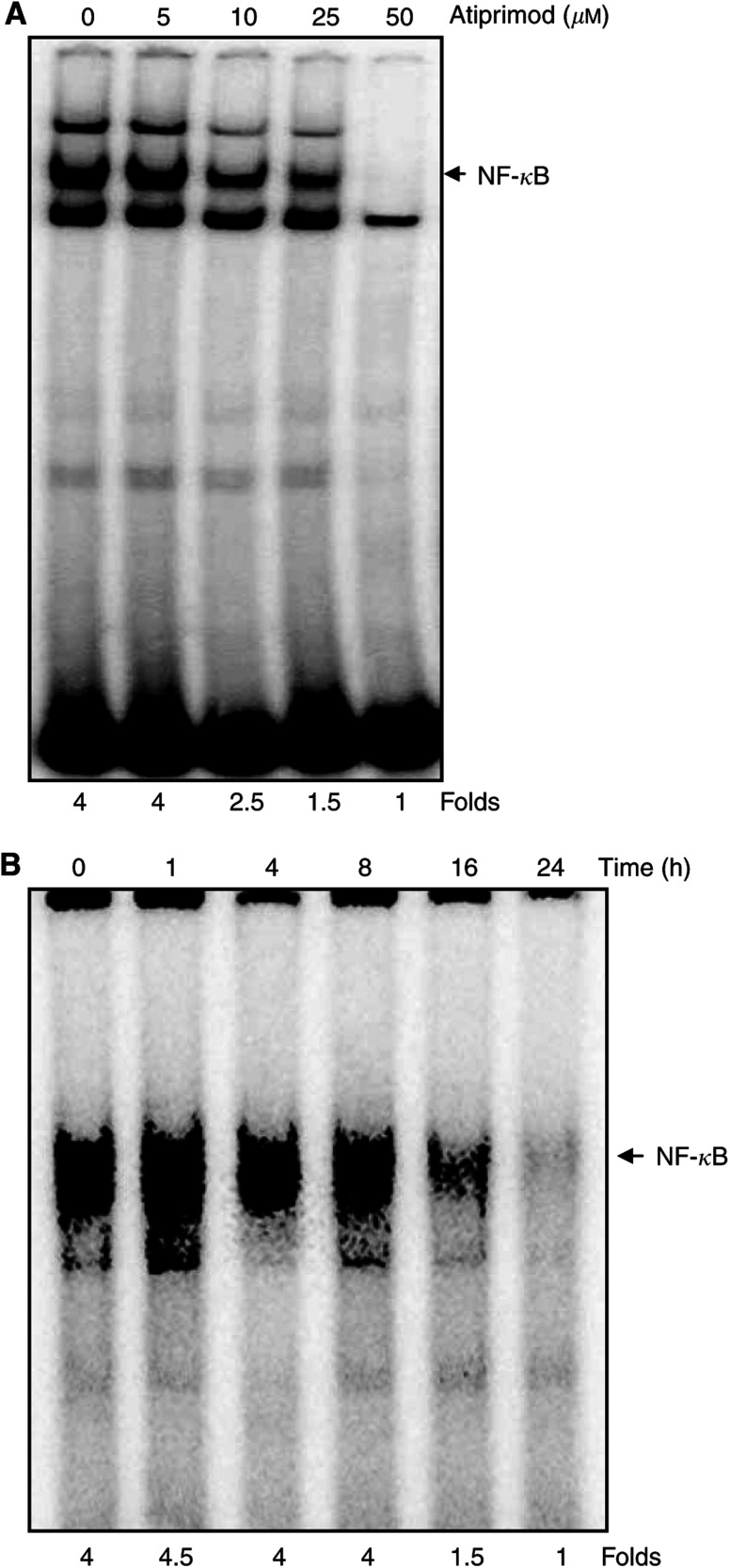
Effect of Atiprimod on activation of NF-*κ*B. U266-B1 cells were incubated for 4 h with increasing concentrations of Atiprimod (**A**) and with 8 *μ*M of Atiprimod for 1, 4, 8, 16, and 24 h (**B**). Nuclear extracts were prepared, and NF-*κ*B activity was analysed by EMSA, as described in Materials and Methods.

Since the expression of IL-6 is regulated by NF-*κ*B (Shimizu *et al*, 1990) and Atiprimod downregulates IL-6 production, we hypothesised that Atiprimod inhibits NF-*κ*B. To test this hypothesis, we used U266-B1 cells, which constitutively express active NF-*κ*B (Ni *et al*, 2001; Bharti *et al*, 2004) and produce IL-6 (Catlett-Falcone *et al*, 1999; Bharti *et al*, 2003). We incubated U266-B1 cells with Atiprimod at increasing concentrations for 4 h, and with 8 *μ*M for 1, 4, 6, 8, and 24 h and tested the NF-*κ*B activity in nuclear extracts. We found that 10 *μ*M but not 5 *μ*M Atiprimod inhibited constitutive NF-*κ*B activity, and that 8 *μ*M Atiprimod inhibited NF-*κ*B activity after a prolonged incubation (16 h) (Figure 1).

### Atiprimod downregulates both IL-6-induced and constitutive STAT3 phosphorylation in MM cells

**Figure 2:**
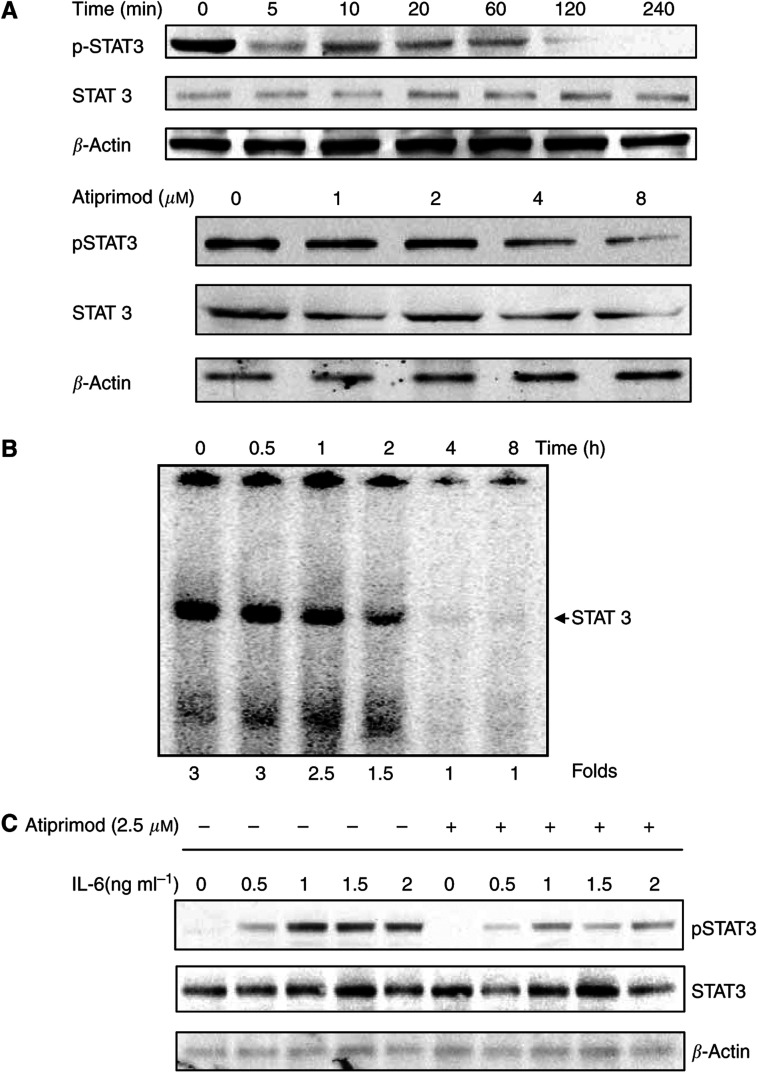
(**A**) Effect of Atiprimod on constitutive STAT3 phosphorylation. U266-B1 (1 × 10^7^ cells ml^−1^) were incubated in RPMI 1640 supplemented with 10% FCS with or without 8 *μ*M Atiprimod for 5–240 min (upper panel). In a separate experiment, 1 × 10^7^ U266-B1 cells were incubated with or without increasing concentrations of Atiprimod for 1 h(lower panel). Signal transducer and activator of transcription (STAT) 3 and phosphorylated (p) STAT3 and were detected by Western immunoblotting as described in Materials and Methods. Equal loading of protein was confirmed by using anti-*β*-actin antibodies. (**B**) Effect of Atiprimod on binding of DNA with activated STAT3. U266-B1 cells were incubated with 8 *μ*M Atiprimod for 0.5, 1, 2, 4, and 6 h. Nuclear extracts were labelled with hSIE probe and STAT3-DNA binding was analysed by EMSA, as described in Materials and Methods. (**C**) Effect of Atiprimod on IL-6-induced STAT3 phosphorylation. MM-1 cells (1 × 10^7^ cells ml^−1^) were incubated for 2 h in RPMI 1640 supplemented with 10% FCS and increasing concentrations of IL-6 with or without 2.5 *μ*M Atiprimod.

Interleukin-6 is produced by MM cells and promotes myeloma cell proliferation in an autocrine manner (Klein *et al*, 1995; Chauhan *et al*, 1996; Jemal *et al*, 2003; Anderson, 2004) through phosphorylation of the signalling protein STAT3 (Kaptein *et al*, 1996; Hirano *et al*, 2000; Chatterjee *et al*, 2002). As Atiprimod inhibits NF-*κ*B activity and, by doing so, suppresses IL-6 production, we wondered whether Atiprimod downregulates STAT3 phosphorylation. Using U266-B1 cells, we found that Atiprimod inhibited STAT3 phosphorylation in a time- and dose-dependent manner (Figure 2A). After U266-B1 cells were incubated for 4 h with 8 *μ*M Atiprimod, the expression of phosphorylated STAT3 protein was reduced to undetectable levels (Figure 2A, upper panel). Similarly, DNA binding of activated STAT3 was reduced by 50% after 2 h and completely abolished after 4 h (Figure 2B). Incubation of U266-B1 cells with Atiprimod at concentrations ranging from 1 to 8 *μ*M for 1 h significantly downregulated STAT3 phosphorylation (Figure 2A, lower panel), suggesting that Atiprimod inhibits the Janus tyrosine kinase (JAK)-STAT signalling pathway in myeloma cells.

Given that Atiprimod inhibited NF-*κ*B activity at a concentration of 10 *μ*M but not 5 *μ*M, and exposure to Atiprimod at a lower concentration (4 *μ*M) for 1 h downregulated STAT3 phosphorylation (Figure 2A), we conclude that Atiprimod inhibited STAT3 phosphorylation directly and that this effect was not mediated through the inhibition of NF-*κ*B.

We then tested the effects of Atiprimod on the IL-6-responsive myeloma cell line MM-1 (Deane and Norton, 1990). As expected, we found that IL-6 induced STAT3 phosphorylation in a dose-dependent manner at concentrations ranging from 0.5 to 2 ng ml^−1^. However, this effect was significantly attenuated by Atiprimod (Figure 2C). These results confirm our previous data showing that that Atiprimod inhibits STAT3 phosphorylation.

### Atiprimod inhibits myeloma cell proliferation

**Figure 3:**
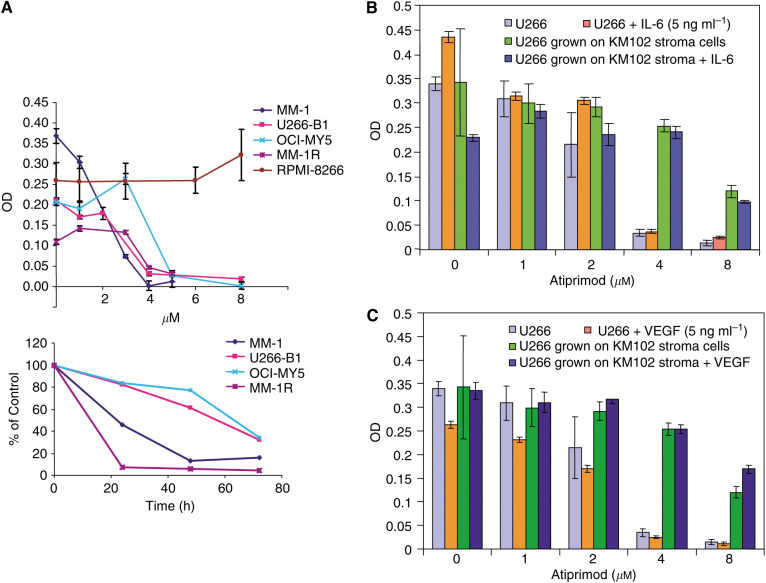
(**A**) Effect of Atiprimod on MM cell lines. Upper panel: MM-1, U266-B1, OCI-M5, MM-1R, and RPMI-8266 (5 × 10^4^) cells were incubated in RPMI 1640 supplemented with 10% FCS with or without increasing concentrations of Atiprimod for 72 h. Cells were harvested and their metabolic activity and viability were determined using the MTT assay. Data are presented as the mean±standard deviation of optical density (OD) measurements of six wells. Lower panel: MM-1, U266-B1, OCI-M5, and MM-1R cells (5 × 10^4^) were incubated with 4 *μ*M Atiprimod for 24, 48, and 72 h. Cells were harvested and their metabolic activity and viability were determined using the MTT assay. Data are presented as a percent of OD of cells cultured without Atiprimod at the same time point (control). (**B**, **C**) Effect of IL-6, VEGF, and BM stroma on Atiprimod-induced inhibition of U266-B1 cells. These cells were cultured alone or with adherent KM102 stroma cells, with or without either IL-6 (**B**) or VEGF (**C**) at 5 ng ml^−1^, and in the presence of increasing concentrations of Atiprimod. After 72 h, U266-B1 cells were harvested and analysed using the MTT assay. The data are presented as the mean±standard deviation of OD measurements of five wells.

As the activation of STAT3 stimulates MM cell proliferation (Kaptein *et al*, 1996; Bharti *et al*, 2004), we sought to determine whether Atiprimod would reduce the proliferation and metabolic activity of different MM cell lines. We found that Atiprimod inhibited U266-B1, OCI-MY5, MM-1, and MM-1R myeloma lines in a dose- and time-dependent fashion (Figure 3A). Atiprimod at a concentration of 5 *μ*M inhibited MM-1 and MM-1R cell growth by 96.7 and 72%, respectively, and Atiprimod at a concentration of 8 *μ*M inhibited U266B-1 and OCI-MY5 cells by 99 and 91.5%, respectively. Atiprimod did not affect the growth of RPMI-8266 myeloma cells.

### The antiproliferative effect of atiprimod is not reversed by IL-6, vascular endothelial growth factor (VEGF), or BM stroma

We next asked whether IL-6, VEGF, or BM stroma, previously shown to promote growth and survival of MM cells (Chauhan *et al*, 1996; Podar *et al*, 2001; Iwasaki *et al*, 2002; Menu *et al*, 2004), would reverse the inhibitory effect of Atiprimod. To investigate this, we plated the BM stroma cell line KM102 in 96-well flat-bottomed plates. After they reached confluence, we added 5 × 10^4^ U266-B1 cells to the wells together with fresh RPMI-1640 supplemented with 10% FCS with or without either IL-6 or VEGF at 5 ng ml^−1^; the cells were then cultured in the presence or absence of Atiprimod for 72 h. After incubation, U266-B1 cells were harvested and assayed using MTT as described above. As was the case in our previous experiments, Atiprimod inhibited U266-B1 cells in a dose-dependent fashion (Figure 3B and C). Although Atiprimod suppressed IL-6 production and either reduced or did not affect soluble IL-6R levels (Tables 2 and 3), neither IL-6 nor VEGF at a concentration of 5 ng ml^−1^ attenuated the inhibitory effect of Atiprimod. Coculture of U266-B1 cells with KM102 stroma cells only partially reversed the inhibitory effect of Atiprimod; the addition of either IL-6 or VEGF to U266-B1 cells cocultured with KM102 cells did not affect Atiprimod’s inhibitory effect (Figure 3B and C). Similar results were obtained with 10 ng ml^−1^ of IL-6 and VEGF (data not shown).

### Atiprimod induces accumulation of MM cells in the sub-G_0_/G_1_ phase of the cell cycle

**Figure 4:**
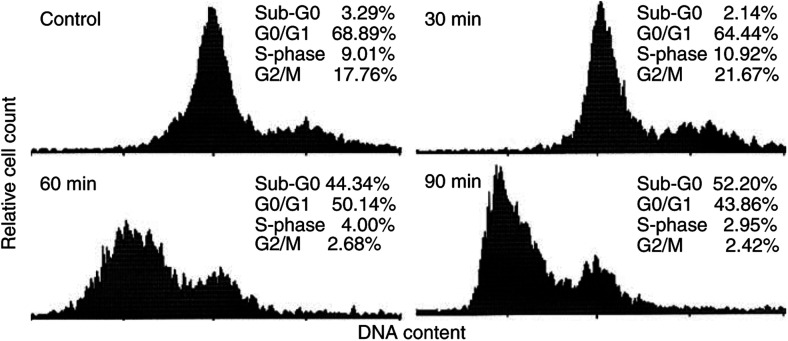
Effect of Atiprimod on the cell cycle status of U266-B1 cells. These cells were incubated in RPMI 1640 supplemented with 10% FCS with or without 6 *μ*M Atiprimod, and cell cycle analysis was performed as described above. The percentages of cells in sub-G_0_, G_0_/G_1_, S, and G2M phases of the cell cycle are given.

As Atiprimod blocked STAT3 phosphorylation and inhibited MM cell proliferation, we asked how this drug affects the progression of U266-B1 cells through the cell cycle. To answer this question, we incubated U266-B1 cells with 6 *μ*M Atiprimod and performed a cell cycle analysis using flow cytometry. We found that Atiprimod induced a sub-G_0_/G_1_accumulation with 44.3 and 52.2% of the cells accumulating in sub-G_0_ phase at 60 and 90 min, respectively (Figure 4).

### Atiprimod downregulates Bcl-2, Bcl-X_L_, and Mcl-1 protein levels

**Figure 5:**
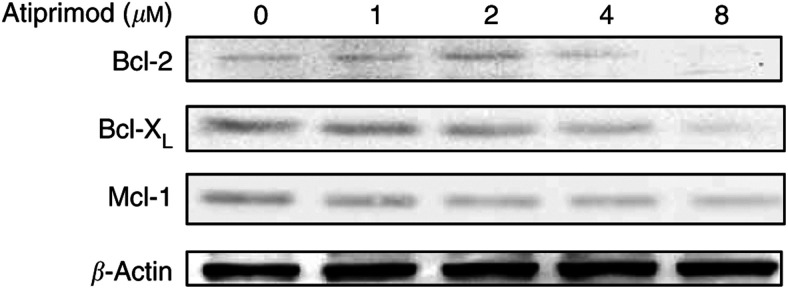
Effect of Atiprimod on Bcl-2, Bcl-X_L_, and Mcl-1 protein levels. U266-B1 cells were incubated for 2 h with Atiprimod at increasing concentrations. The levels of Bcl-2, Bcl-X_L_, and Mcl-1 were detected by Western immunoblotting. The results shown here demonstrate a dose-dependent decrease in the levels of Bcl-2, Bcl-X_L_, and Mcl-1.

The Bcl-2 protein family is involved in the regulation of apoptotic cell death. Recent studies showed that several myloma cell lines express Bcl-2 protein at high levels (Pettersson *et al*, 1992), providing MM cells with survival advantage (Feinman *et al*, 1999), and that activation of STAT3 upregulates the expression of Bcl-X_L_ (Catlett-Falcone *et al*, 1999). Furthermore, IL-6 was shown to upregulate Mcl-1 levels in MM cells through activation of the JAK-STAT pathway (Puthier *et al*, 1999). Therefore, we asked whether Atiprimod would affect the levels of Bcl-2, Bcl-X_L_, and Bcl-1. As shown in Figure 5, we found that Atiprimod reduced the levels of these proteins in U266-B1 cells.

### Atiprimod induces apoptotic cell death

**Figure 6:**
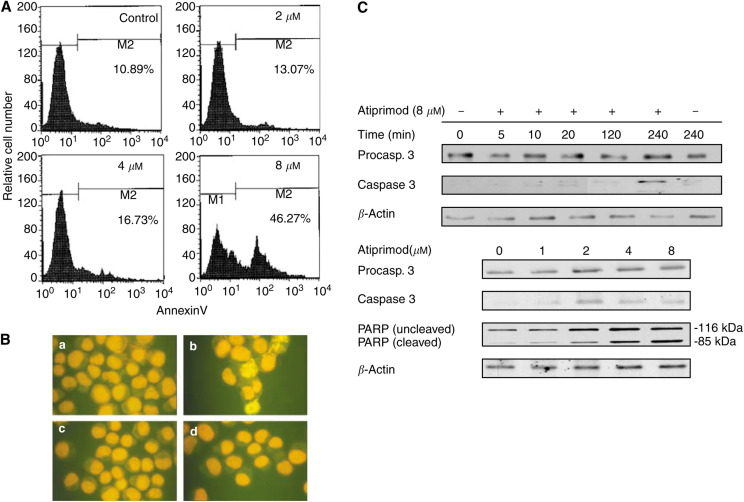
Atiprimod induces apoptosis in myeloma cells. (**A**) U266-B1 cells were incubated with or without 2, 4, or 8 *μ*M Atiprimod for 4 h. The fraction of cells undergoing apoptosis was detected by annexin V-CY5. The percentages in each frame indicate the apoptotic cell fraction. (**B**) U266-B1 cells were incubated with (b, d) or without (a, c) 8 *μ*M Atiprimod and with (c, d) or without (a, b) 50 *μ*M Ac-DEVD-CHO. After incubation, cytospun cells were stained with TUNEL. Yellow-appearing cells (apoptotic cells) were detected after exposure to Atiprimod (b). The addition of Ac-DEVD-CHO, which by itself did not affect cell morphology (c) reversed this process (d). (**C**) Atiprimod activates caspase 3 and cleaves PARP. U266-B1 cells were incubated with 8 *μ*M Atiprimod for 0, 5, 10, 20, 120, and 240 min (upper panel) and Atiprimod at increasing concentrations for 1 h (lower panel). The levels of procaspase 3, caspase 3, and uncleaved and cleaved PARP were detected by Western immunoblotting. The results shown here demonstrate a time-dependent increase in the cleaved caspase 3 level (upper panel) and a dose-dependent increase in the cleaved caspase 3 and cleaved PARP levels (lower panel).

As Atiprimod blocked STAT3 activation, inhibited MM cell proliferation, induced cell cycle arrest in U266-B1 cells at the G_0_/G_1_ phase, and reduced the levels of Bcl-2, Bcl-X_L_, and Mcl-1, we hypothesised that Atiprimod induces apoptotic cell death. To test this hypothesis, we incubated U266-B1 cells at the peak of their growth for 4 h in the presence or absence of 2, 4, or 8 *μ*M Atiprimod. Using annexinV-CY5, we demonstrated that Atiprimod induced apoptosis in U266-B1 cells in a dose-dependent fashion. The percentage of cells undergoing apoptotic cell death increased from 10.89 to 46.27% after exposure to 8 *μ*M Atiprimod (Figure 6A). Interestingly, U266-B1 cells accumulated in sub-G_0_ phase of the cell cycle after 60 (44.3%) and 90 (52.2%) min of exposure to 6 *μ*M Atiprimod (Figure 4). Thus, either not all cells accumulated in sub-G_0_ phase undergo apoptosis or different apoptosis assays yield dissimilar results (Sailer *et al*, 1988; Clarke *et al*, 2000).

### Atiprimod induces apoptosis by activating the caspase pathway

To validate these findings, we incubated U266-B1 cells with or without 8 *μ*M Atiprimod in the presence or absence of 50 *μ*M of the caspase inhibitor Ac-DEVD-CHO and used the TUNEL assay to detect apoptotic cells. As shown in Figure 6B, we found that Atiprimod induced apoptotic cell death in 90% of U266-B1 cells, and the addition of c-DEVD-CHO blocked Atiprimod-induced apoptosis.

Apoptosis is executed through the activation of a family of cystein proteases named caspases, which are synthesised as latent intracellular proenzymes. Cleavage of the procaspase forms converts them into biologically active caspases (Strasser *et al*, 2000; Orlowski and Baldwin, 2002). We chose to study the effect of Atiprimod on the activation of caspase 3, the downstream ‘executioner’ caspase (Nicholson, 1999) in U266-B1 cells. As shown in the Western immunoblot in the upper panel of Figure 6C, Atiprimod induced procaspase 3 cleavage in a time-dependent manner, with the maximum effect achieved after 4 h. Furthermore, incubation of U266-B1 cells for 1 h with Atiprimod induced a dose-dependent increase in caspase 3 cleavage (Figure 6C, lower panel).

Activated caspase 3 abrogates the effect of substrates that protect cellular integrity, such as the DNA-repair enzyme PARP (Nicholson, 1999; Kraus and Lis, 2003). When we exposed the cells to Atiprimod, we found a dose-dependent increase in cleaved PARP protein levels, with the maximum effect achieved at a concentration of 8 *μ*M (Figure 6C, lower panel).

### Atiprimod inhibits MM colony-forming cell proliferation

**Figure 7:**
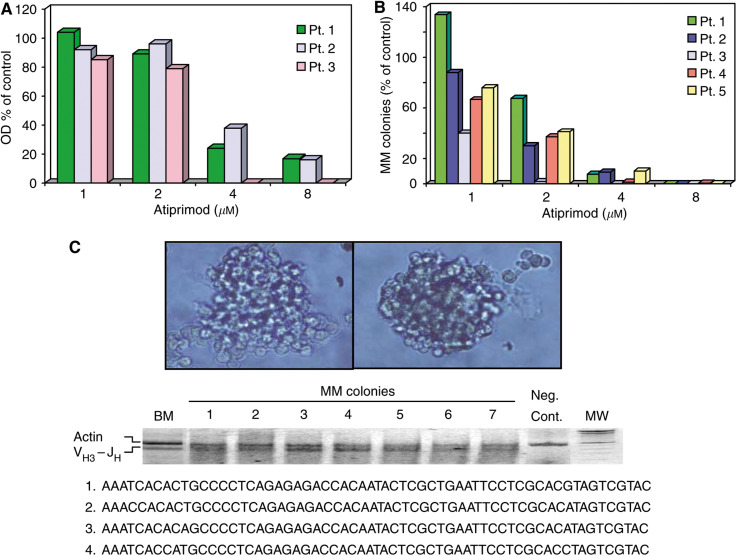
Atiprimod inhibits primary MM cell proliferation. (**A**) Low-density BM cells from three patients with MM and incubated in the presence of Atiprimod at concentrations ranging from 1 to 8 *μ*M and analysed using the MTT assay. (**B**) The effect of Atiprimod on MM colony-forming cells was studied on marrow cells from five patients with MM. After fractionation, myeloma cells were cultured in the presence of Atiprimod at concentrations ranging from 1 to 8 *μ*M. Multiple myeloma colonies are presented as percentages of control (the mean number of colonies obtained from patients 1 through 5 in the absence of Atiprimod was 82, 243, 133, 179, and 467, respectively). (**C**) The top panel depicts two typical MM colonies. Of note are the large cells with myeloma cell morphology at the periphery of the colony. The middle panel shows the products of PCR amplification of DNA from the original marrow of patient 2 and from seven single colonies microaspirated at random using V_H3_–J_H_ consensus primers. The myeloid leukaemia cell line OCIM2 was used as a negative control (Neg. Cont.). Actin was used as an amplification control (MW denotes molecular weight). The lower panel shows the nucleotide sequences of amplified products of DNA from the D region of the diagnostic marrow (1) and of three different colonies (2–4). The middle and lower panels demonstrate that the MM colonies originated from the neoplastic clone.

In light of these results, we postulated that Atiprimod would also inhibit the proliferation of primary MM cells. To investigate this, we first studied the effect of Atiprimod on fresh low-density marrow cells from three patients with MM. We incubated the cells with increasing concentrations of Atiprimod and analysed its effect using the MTT assay. As shown in Figure 7A, we found that Atiprimod suppressed the metabolic activity of MM cells in a dose-dependent manner with no activity detected at 8 *ì*M. We then asked whether Atiprimod would also inhibit myeloma colony-forming cells. We obtained BM cells from five patients with newly diagnosed, untreated MM (Table 1). As depicted in Figure 7B, we found that Atiprimod suppressed MM colony-forming cell growth in a dose-dependent manner, reaching 85% suppression at 4 *μ*M and 100% at 8 *μ*M.

Multiple myeloma colonies were identified by their morphological characteristics, as depicted in the upper panel of Figure 7C. To verify that the MM colony-forming cells originated from the neoplastic clone, single colonies were microaspirated at random, and the rearranged IgH gene in DNA from the original BM sample and from individual MM colonies was amplified via PCR using primers chosen from the first framework region of the coding strand of representative germline V_H_ family members and a J_H_ consensus primer. As shown at the bottom of Figure 7C, all analysed colonies showed an IgH gene rearrangement identical to that of the diagnostic marrow cells. Sequence analysis of colony-derived DNA showed a 90% homology with the original MM cell DNA, confirming the neoplastic clonal origin of the colony-forming cells.

## DISCUSSION

Several growth factors and cytokines stimulate MM cell proliferation by activating various signalling pathways (Zhang *et al*, 2003; Giuliani *et al*, 2004). Of those molecules, IL-6 has been studied extensively, and its pathophysiologic effects in MM have been well characterised (Klein *et al*, 1995; Chauhan *et al*, 1996). Since Atiprimod downregulates serum IL-6 levels (Bradbeer *et al*, 1996), we tested Atiprimod's effect on U266-B1 cells, known to constitutively produce IL-6 (Catlett-Falcone *et al*, 1999), and found that Atiprimod suppressed IL-6 production in a time-dependent manner.

Since IL-6 expression is regulated by NF-*κ*B (Shimizu *et al*, 1990), we investigated whether Atiprimod inhibits NF-*κ*B activity. The NF-*κ*B transcription factor family is an important modulator of cellular proliferation, suppression of apoptosis, enhancement of tumour cell invasiveness, and induction of angiogenesis (Orlowski and Baldwin, 2002). The antitumour activity of the proteasome inhibitor bortezomab in MM is thought to be mediated through the inhibition of NF-*κ*B (Voorhees *et al*, 2003). We recently found that fresh BM cells obtained from MM patients express constitutively active NF-*κ*B and STAT3 (Bharti *et al*, 2004). Furthermore, MM patients’ myeloma cells constitutively produce IL-6 (Catlett-Falcone *et al*, 1999; Bharti *et al*, 2003). Thus, it is possible that constitutively activated NF-*κ*B induces the production of IL-6, which in turn activates STAT3 (Kaptein *et al*, 1996; Chatterjee *et al*, 2002) and MM cell proliferation. Therefore, we wondered whether Atiprimod inhibits NF-*κ*B activity and, by doing so, downregulates constitutive production of IL-6. Using U266-B1 cells, which express constitutively active NF-*κ*B and STAT3 (Bharti *et al*, 2004) and produce IL-6, we found that Atiprimod inhibited NF-*κ*B activation.

Since Atiprimod inhibited NF-*κ*B and IL-6 production, we wondered whether Atiprimod also downregulated IL-6 signalling. Using U266-B1 cells, we found that Atiprimod inhibited STAT3 phosphorylation after a shorter incubation and at concentrations lower than those required for the inhibition of NF-*κ*B. As these results suggested that Atiprimod directly inhibited STAT3 phosphorylation, we used MM-1 cells in which the JAK-STAT pathway is activated upon exposure to exogenous IL-6 (Chatterjee *et al*, 2002). As in our previous experiments with U266-B1 cells, we found that Atiprimod inhibited the IL-6-induced STAT3 phosphorylation in MM-1 cells in a time- and dose-dependent manner. These results suggest that Atiprimod suppresses STAT3 phosphorylation directly, and that this effect is not dependent on the inhibition of NF-*κ*B. Nevertheless, the ability of Atiprimod to inhibit NF-*κ*B, a proven target in myeloma treatment, suggests that Atiprimod might also inhibit myeloma cells through that mechanism, provided that higher concentrations of this drug are used.

As Atiprimod blocked STAT3 phosphorylation and thus the JAK-STAT pathway, we tested the effect of Atiprimod on MM cell proliferation. As expected, Atiprimod inhibited several myeloma cell lines. Specifically, Atiprimod induced accumulation of myeloma cells at the sub-G_0_/G_1_ phase of the cell cycle. Atiprimod inhibited MM-1 and the dexamethasone-resistant MM-1R cells in the absence of exogenous IL-6. Although small amounts of IL-6 present in FCS could have stimulated the JAK-STAT pathway in these cells, Atiprimod might have blocked other signalling pathways involved in stimulating the proliferation of MM-1 and OCI-MY5 cells.

We then asked whether Atiprimod could inhibit the proliferation of primary MM cells. Using the myeloma colony culture assay, we found that Atiprimod suppressed the growth of myeloma colony-forming cells of fresh BM samples obtained from five patients with newly diagnosed MM in a dose-dependent fashion.

As discussed above, several factors besides IL-6 contribute to myeloma cell proliferation. In particular, VEGF and marrow stroma are major players in this process (Chauhan *et al*, 1996; Anderson, 1999; Podar *et al*, 2001; Iwasaki *et al*, 2002).

VEGF stimulates MM cells by triggering activation of the JAK-STAT pathway (Rabinovitch *et al*, 1993) and exerts a proangiogenic effect that is thought to play a role in the pathogenesis of MM (Anderson, 1999). Interestingly, the levels of VEGF have been found to correlate with disease stage and progression (Iwasaki *et al*, 2002). Bone marrow stroma also contributes to the myelomatous process through the production of stimulating cytokines (Chauhan *et al*, 1996). Additionally, according to a recent study, MM cells become independent of the STAT3 pathway in the presence of BM stroma (Chatterjee *et al*, 2002). Therefore, we sought to determine whether IL-6, VEGF, and BM stroma interfere with the antiproliferative effect of Atiprimod. We found, however, that neither IL-6 nor VEGF negated the effect of Atiprimod, and that the BM stroma cell line KM102 only partially reversed Atiprimod's inhibitory effect.

The activation of STAT3 in myeloma cells results in the upregulation of antiapoptotic proteins of the Bcl-2 family, such as Bcl-2, Bcl-X_L_, and Mcl-1, thus providing MM cells with a survival advantage (Pettersson *et al*, 1992; Catlett-Falcone *et al*, 1999; Feinman *et al*, 1999; Puthier *et al*, 1999). As Atiprimod suppresses STAT3 phosphorylation, we asked how it would affect Bcl-2, Bcl-X_L_, and Mcl-1 protein levels. We found that Atiprimod downregulated the levels of Bcl-2, Bcl-X_L_, and Mcl-1, and induced apoptotic cell death in U266-B1 myeloma cells. Atiprimod's proapoptotic effect was blocked by the caspase inhibitor Ac-DEVD-CHO, suggesting that apoptosis was induced through the activation of the caspase pathway. Indeed, exposure of U266-B1 myeloma cells to Atiprimod resulted in the activation of the downstream executioner caspase 3 and subsequent cleavage of the DNA repair enzyme PARP.

Taken together, our data show that Atiprimod blocks STAT3 phosphorylation and the activation of NF-κB, inhibits cellular proliferation, causes cell cycle arrest, and induces apoptosis in MM cells. As Atiprimod was well tolerated in patients with rheumatoid arthritis, phase I studies of Atiprimod in patients with MM are warranted.

## Figures and Tables

**Table 1:** Clinical data on MM patients

**Patient**	**Age/sex**	**MM Type**	**Hb (g dl^−1^)**	**WBC (× 10^9^ l^−1^)**	**Platelets (× 10^9^ l^−1^)**	**% BM myeloma cells**	**Serum paraprotein (g dl^−1^)**	**Urine paraprotein (g 24 h^−1^)**
1	44/F	IgG	9.4	10.3	322	55	6.1	1.0
2	48/F	IgA	6.9	6.0	45	70	3.5	0.4
3	56/M	IgG	12.4	5.9	254	36	3.4	0.16
4	48/M	IgG	10.1	13.2	231	58	6.8	1.2
5	59/M	IgA	12.5	4.8	181	44	3.4	0.1

F=female; M=male; MM=multiple myeloma; Hb=haemoglobin; WBC=white blood cells; BM=bone marrow.

**Table 2:** IL-6 supernatant levels (pg ml**^−1^**)

			**+ Normal stroma**		**+KM102**	
	−	**Atiprimod**	−	**Atiprimod**	−	**Atiprimod**
Normal stroma	650	467				

KM102	521	485				

MM1	0	0	573	594	583	566

MM1R	0	0	572	524	271	294

U266-B1	227	56	642	514	645	354

OCI-MY5	0	0	714	632	731	686

Cells were incubated for 72 h with or without 3 *μ*M Atiprimod. The means of interleukin-6 (IL-6) levels from duplicate wells are depicted.

**Table 3:** Soluble interleukin-6 receptor (IL-6R) supernatant levels (pg ml^−1^)

			**+Normal stroma**		**+KM102**	
	−	**Atiprimod**	−	**Atiprimod**	−	**Atiprimod**
Normal stroma	650	467				

KM102	521	485				
MM1	0	0	573	594	583	566
MM1R	0	0	572	524	271	294
U266-B1	27	0	642	514	645	354
OCI-MY5	0	0	714	632	731	686

Cells were incubated for 72 h with or without 3 *μ*M Atiprimod. The means of soluble IL-6R levels from duplicate wells are depicted.
